# IL-27: overclocking cytotoxic T lymphocytes to boost cancer immunotherapy

**DOI:** 10.1038/s41392-025-02212-z

**Published:** 2025-04-21

**Authors:** Silvia Dusi, Vincenzo Bronte, Francesco De Sanctis

**Affiliations:** 1 Veneto Institute of Oncology IOV-IRCCS, Padua, Italy; 2 Department of Medicine, Section of Immunology, University of Verona, Verona, Italy

**Keywords:** Tumour immunology, Immunotherapy

In a recent study published in *Nature*, Breart et al.^1^ untangled the controversy surrounding interleukin (IL)-27’s role in regulating T cell differentiation and immune responses in cancer. Their findings reveal that IL-27 enhances the persistence and effector functions of tumor-infiltrating CD8^+^ T lymphocytes (TILs), preventing exhaustion, sustaining T cell-dependent tumor control and immunotherapy efficacy without significant systemic side effects.

Effective immune control of cancer relies on the ability of T lymphocytes to infiltrate tumors and exert their cytotoxicity. Nonetheless, surviving neoplastic cells promptly adapt to immune-patrolled environments and evolve under immune system pressure to progressively avoid immune clearance, establishing immune-privileged conditions that sustain limitless growth. These immune evasive strategies are deployed in 3 main routes: “camouflage” (reduced immunogenicity and immune exclusion), “coercion” (impaired local immune activation through the establishment of a highly immunosuppressive tumor microenvironment [TME]), “cytoprotection” (avoiding neoplastic cell killing through intrinsic and extrinsic resistance mechanisms).^2^ Therapeutic approaches targeting immune checkpoints and aiming to restore cancer immune surveillance significantly improved the survival and life quality of cancer patients, although their efficacy is restricted to a fraction of patients and tumor types.

**Fig. 1:**
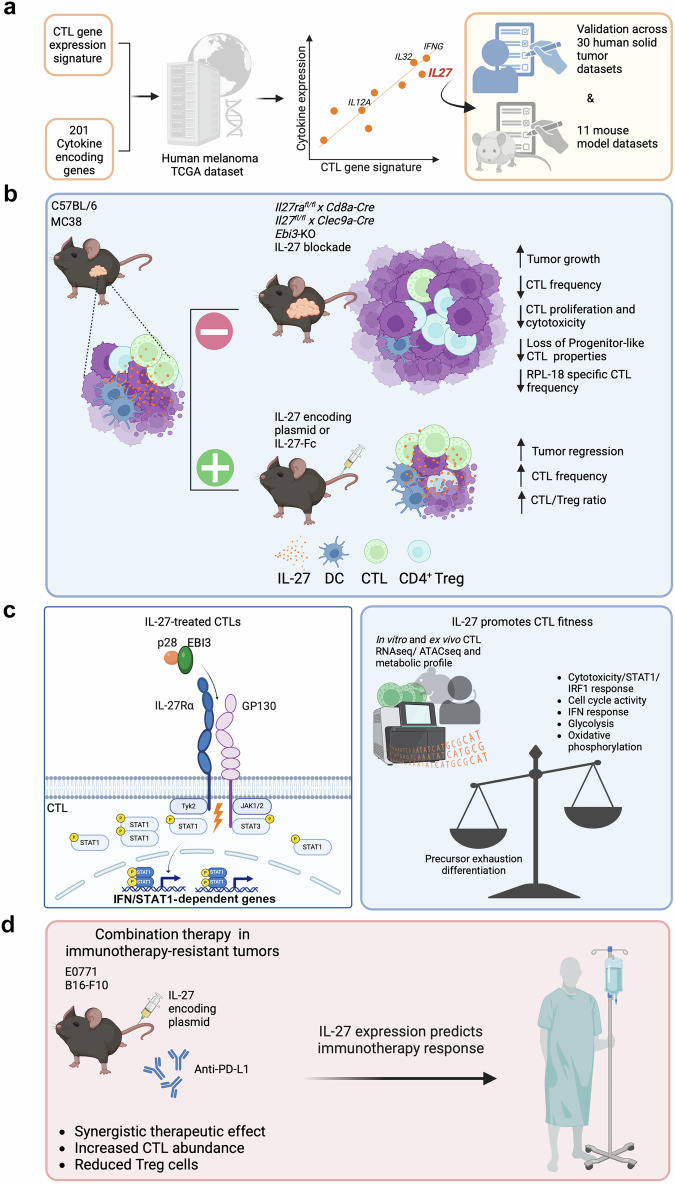
Unveiling IL-27 association with CTL infiltration and characterization of its mechanism of action. **a** Cytokine genes were correlated with a CTL signature in a human melanoma dataset to identify genes promoting lymphocyte infiltration. The significant, positive IL-27 association with CTLs was validated in 41 mouse and human different solid tumor datasets. **b** Blocking IL-27-IL-27R axis or IL-27R agonism either reduces or promotes T cell infiltration in tumor and tumor control, respectively. **c** IL-27 triggers STAT1/3, activating an IFN response that promotes effector functions, and metabolic fitness while dampening the exhaustion program. **d** IL-27 ectopic expression restores and strengthens ICI efficacy in poorly responsive preclinical tumor models; pretreatment *IL-27* expression is prognostic of better response to ICI in different solid tumors. The figure was created using BioRender

Breart et al. attempted to identify novel, actionable immunological targets by charting the cytokines, which are known to be the most potent immune system regulators. By correlating a gene signature of cytotoxic T lymphocyte (CTL) infiltration and activity with the expression of more than 200 cytokine genes in human and mouse tumor RNA-seq datasets, they identified a strong correlation with interferon-gamma and interleukin (IL)27 (Fig. 1a). IL-27 has been associated with improved T helper 1 response and attenuated tumor growth in previous studies. However, its role in CTL regulation has remained controversial since IL-27 can also induce the upregulation of markers associated with T cell exhaustion and dysfunction, including PD1 and CD39. Integrating engineered mouse models with molecular studies and functional experiments, the authors solved this apparent paradox, and proved the essential role of IL-27 in CTL infiltration, fitness, and tumor control.

Single-cell RNA-seq confirmed that IL-27 is expressed by myeloid cells, including tumor-associated macrophages (TAMs) and dendritic cells (DCs), and acts on all lymphoid cells that express IL-27R, including T and B lymphocytes as well as natural killers (NKs). In an immunogenic model of colon tumor (MC38), the authors demonstrated that IL-27 blockade promoted tumor progression. Similar results were achieved when *IL-27* was deleted in DCs or *IL27ra* was knocked-out in CTLs, demonstrating the relevance of DC-derived IL-27 in supporting T cell-dependent antitumor activity. Conversely, IL-27R agonism through hydrodynamic tail vein injection of IL-27-encoding plasmid resulted in complete tumor control without relevant toxicity, a common side effect associated with cytokine-based therapies (Fig. 1b). The authors extended this analysis to breast (E0771) and skin (B16-F10) syngeneic tumor models, which are poorly responsive to immune checkpoint inhibitors (ICI), and demonstrated that IL-27 was not effective as single treatment but synergized with ICI to control or delay tumor progression.

To dissect IL-27-driven immune-mediated responses, the authors further extended their research to the MC38 cell line expressing an H2-K^b^-restricted neoantigen (ribosomal protein L18 – RPL18). IL-27 ectopic expression completely reshaped the TME by promoting overall and RPL18-specific CTL infiltration in the tumor, as well as enhancing their cytotoxic activity, as demonstrated by IL-27-dependent upregulation of Ki-67, granzyme B, PD1, CD39, and SCA1 markers. The authors performed an unbiased molecular characterization of RPL18-specific CTLs isolated from either MC38 tumors or draining lymph nodes of mice treated with IL-27 encoding plasmid, empty control plasmid, or IL-27 blocking antibody. IL-27 expression induced cytotoxic and proliferative programs and impaired transition to an exhausted phenotype, without altering the TCR repertoire. Metabolic and epigenetic profiling confirmed that IL-27 triggered STAT1/STAT3 pathways and induced IRF1 expression. These transcription factors enhanced CTL metabolic fitness, and sustained their cytotoxic performances (PD1, GZMB) under exhaustion-induced conditions (Fig. 1c). Similar results were achieved with human CTLs engineered with a transgenic TCR recognizing cytomegalovirus antigen.

Beart et al. aimed to translate these fundamental discoveries in a novel therapeutic approach that could be promptly extended to the clinic. Since cytokines have short in vivo half-lives, the research team engineered IL-27 as a fusion protein with an IgG2a Fc domain. This chimeric protein showed comparable biochemical properties in inducing STAT1/STAT3 activation but superior half-life in vivo, and triggered a dose-dependent MC38 tumor regression without evident toxicity. Notably, IL-27Fc-dependent tumor control was abrogated in mice with CTL-restricted IL-27RA deficiency. In agreement with these results, the baseline *IL-27* expression in tumors predicted response to anti-PD-L1 immunotherapy (but not chemotherapy) in metastatic urothelial bladder carcinoma and non-small cell lung cancer patients (Fig. 1d).

IL-27R expression is shared across multiple lymphoid subsets, including T helper, CTLs, B lymphocytes, and NKs; IL-27 induces the polarization of Th1 responses and effector CTL program, as well as activates T follicular helper cells and B lymphocytes.^3^ Whether IL-27 could either promote or restrict CD8^+^ T lymphocyte exhaustion and sustain or dampen antitumor immunity was still debated. While the IL-27-driven mechanism of uncoupling the effector functions from the exhaustion program requires further characterization, Beart et al. demonstrated that IL-27R agonism may strengthen immunotherapy efficacy in immunogenic tumor models. Future studies should explore whether IL-27 could overcome immunotherapy resistance in solid tumors with low immunogenicity and highly suppressive T cell hostile TME; however, recent evidence suggests that IL-27 can skew TAM polarization towards an M1-like phenotype^4^ and it is becoming evident that reshaping TME towards an antitumor milieu is pivotal to promote immunotherapy efficacy.^5^ However, baseline IL-27 expression predicted immunotherapy response in only a limited number of datasets and tumor types. Therefore, it is essential to expand this analysis to additional solid tumors where immunotherapy is a standard treatment. Further investigation, potentially in combination with other gene candidates, is necessary to optimize therapeutic strategies and ensure the most effective treatment selection for patients.

In conclusion, the authors’ findings highlight the potential of targeting the IL-27–IL-27RA axis to enhance ICI efficacy in cancer. Given the widespread expression of IL-27R on both immune and non-immune cells, as well as IL-27’s dual effects on tumor cells, further research is needed to unveil its role in cancer patients. In this context, an IL-27-targeting antibody (SRF-388/CHS-388) has been developed, and three recent clinical trials are currently evaluating the safety and efficacy of IL-27 blockade, with or without immunotherapy, across various solid tumors. Notably, phase 2 trials are assessing whether IL-27 inhibition can enhance the efficacy of Bevacizumab plus Atezolizumab (NCT05359861) or Bevacizumab plus Toripalimab (NCT06679985) in patients with advanced or metastatic hepatocellular carcinoma.

These findings could pave the way for novel therapeutic strategies harnessing IL-27 to improve cancer immunotherapy outcomes.
